# Supplementary material for: DMRforPairs: identifying Differentially Methylated Regions between unique samples using array based methylation profiles
Source: BMC Bioinformatics. 2014 May 15;15:141. doi: 10.1186/1471-2105-15-141 (PMC4046028; doi:10.1186/1471-2105-15-141)
Supplement: Additional file 2 — DMRforPairs output for the comparison of A431-MCF7 and NA17018-NA17105. Please start from the HTML files in each folder. Available via the BMC Bioinformatics website. [file 1471-2105-15-141-S2.zip › 1394847754114233_MOESM2_ESM/A431_MCF7/figures/22960.pdf]

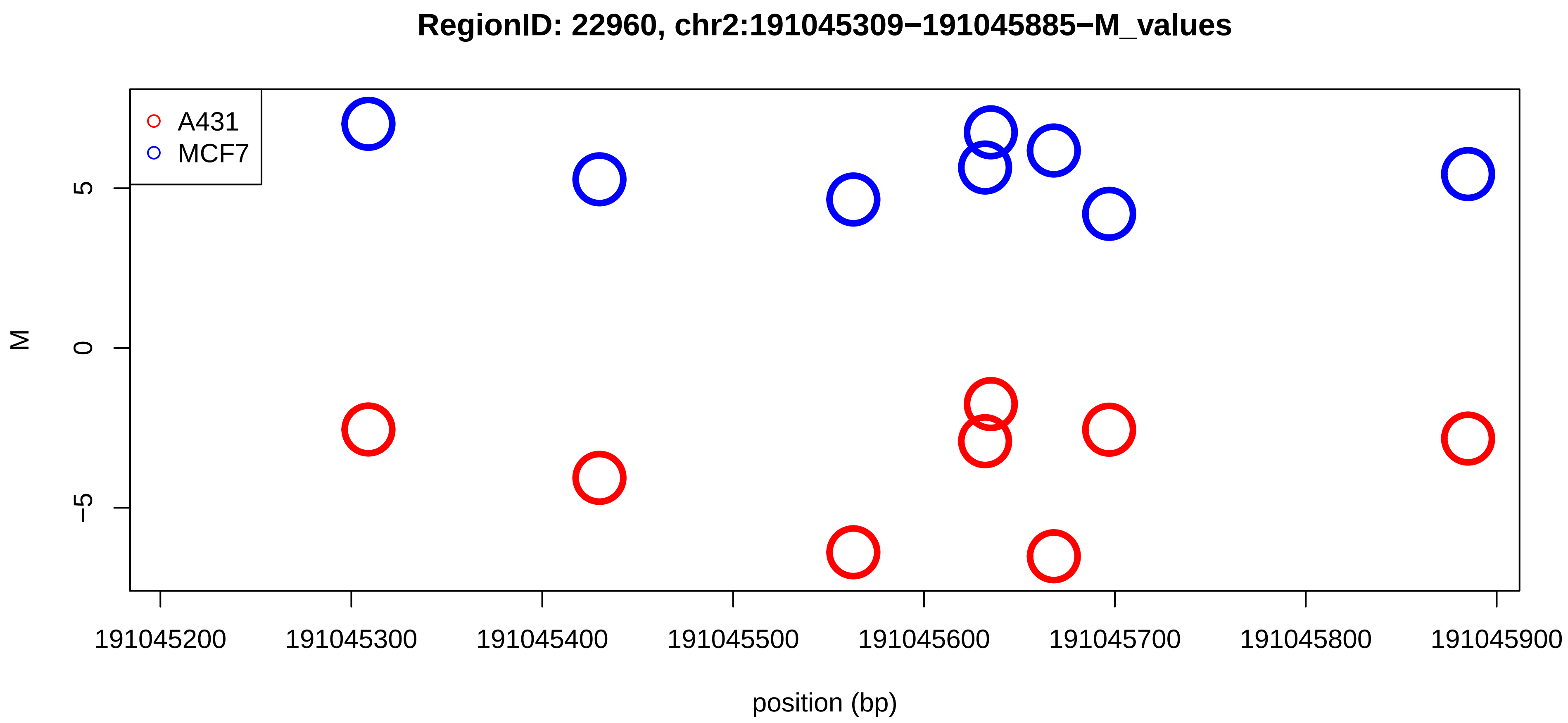

RegionID: 22960, chr2:191045309−191045885−M_values
A431
MCF7
M
5
0
−5
191045200
191045300
191045400
191045500
191045600
191045700
191045800
191045900
position (bp)

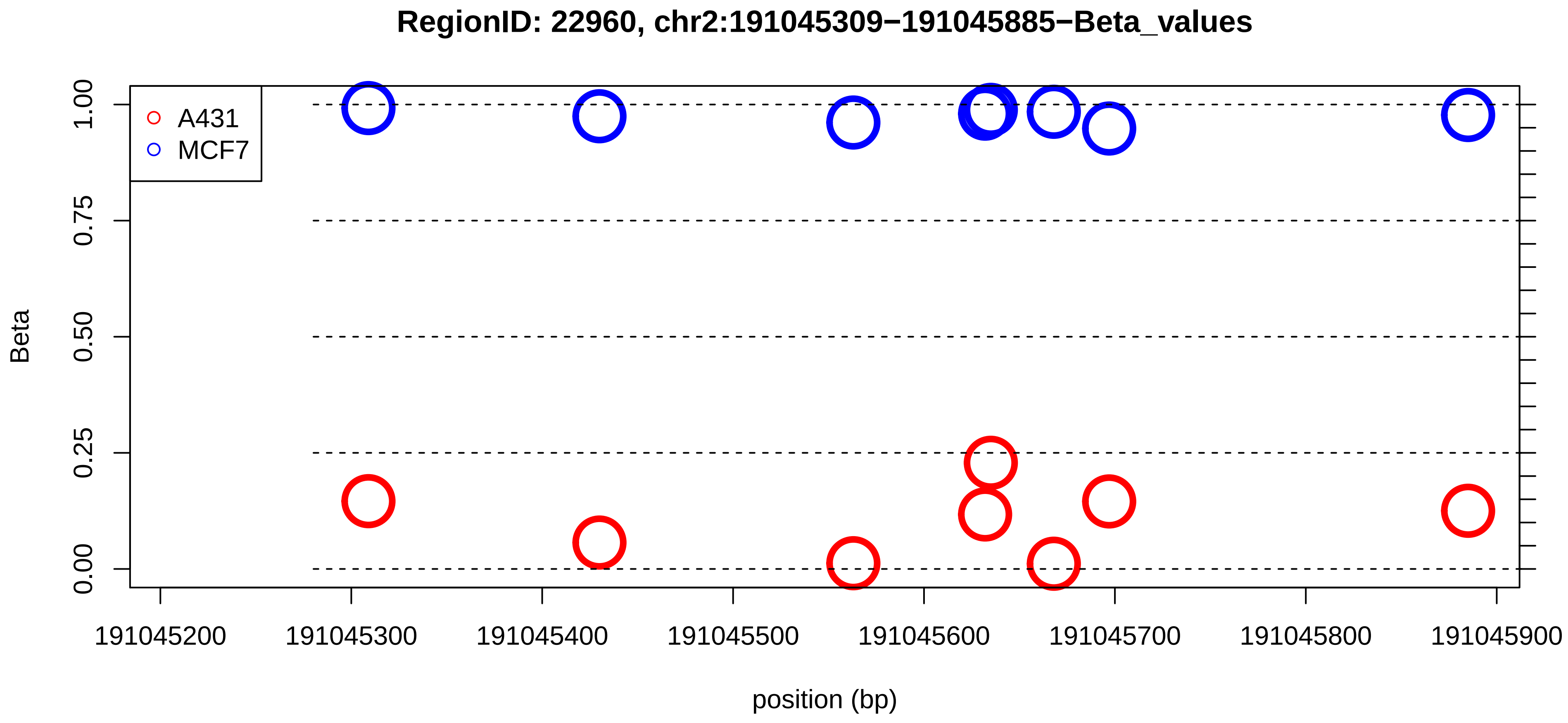

RegionID: 22960, chr2:191045309−191045885−Beta_values
A431
MCF7
Beta
1.00
0.75
0.50
0.25
0.00
191045200
191045300
191045400
191045500
191045600
191045700
191045800
191045900
position (bp)

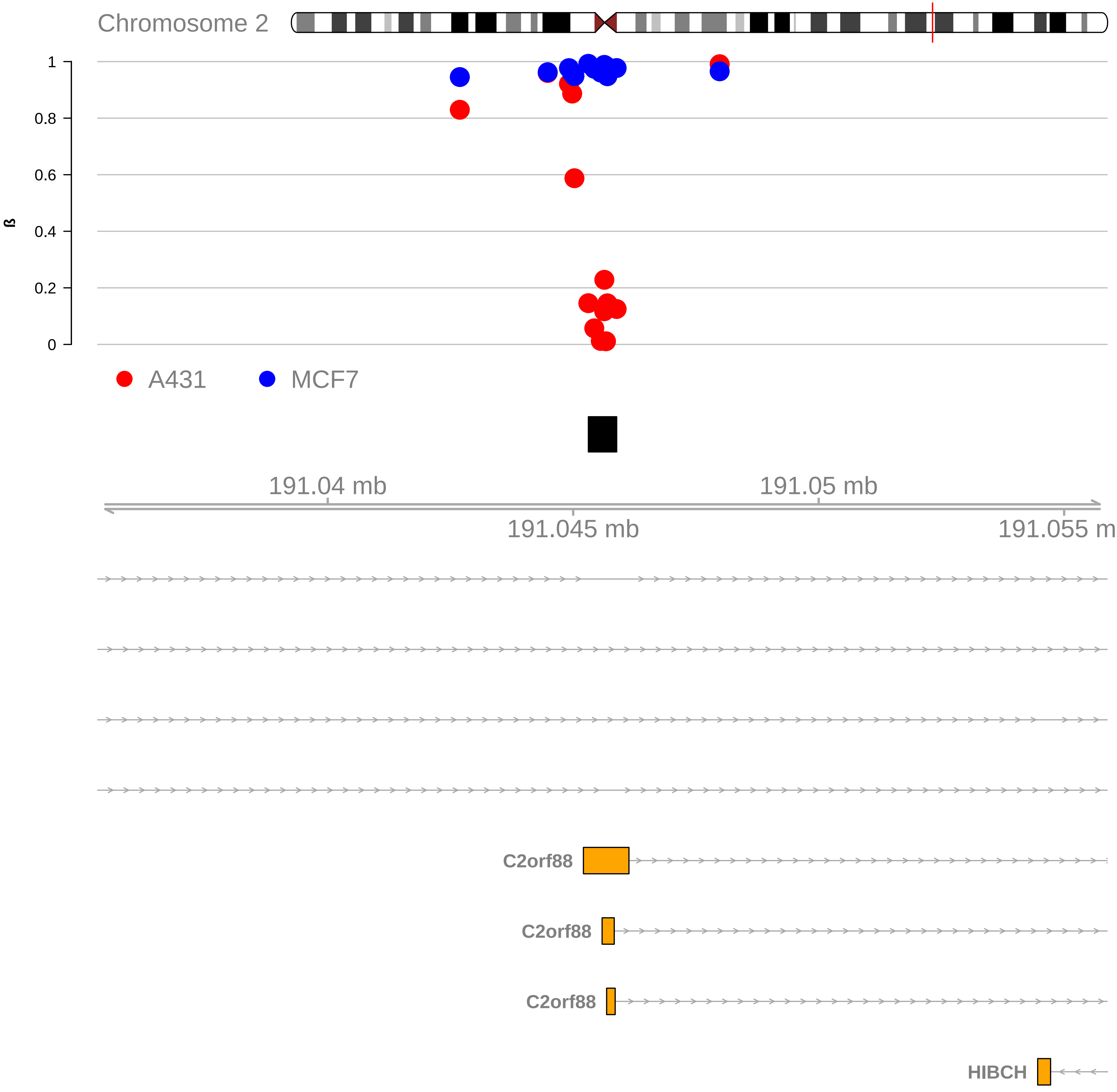

Chromosome 2
β
1
0.8
0.6
0.4
0.2
0
A431
MCF7
191.04 mb
191.05 mb
191.045 mb
191.055 m
C2orf88
C2orf88
C2orf88
HIBCH
